# Genome-Wide Association Study of Maternal and Inherited Loci for Conotruncal Heart Defects

**DOI:** 10.1371/journal.pone.0096057

**Published:** 2014-05-06

**Authors:** A. J. Agopian, Laura E. Mitchell, Joseph Glessner, Angela D. Bhalla, Anshuman Sewda, Hakon Hakonarson, Elizabeth Goldmuntz

**Affiliations:** 1 Human Genetics Center, Division of Epidemiology, Human Genetics and Environmental Sciences, University of Texas School of Public Health, Houston, Texas, United States of America; 2 Department of Pediatrics, University of Pennsylvania Perelman School of Medicine, Philadelphia, Pennsylvania, United States of America; 3 Division of Cardiology, The Children's Hospital of Philadelphia, Philadelphia, Pennsylvania, United States of America; 4 Center for Applied Genomics, The Children's Hospital of Philadelphia, Philadelphia, Pennsylvania, United States of America; Center for Human Genetics, Germany

## Abstract

Conotruncal and related heart defects (CTDs) are a group of serious and relatively common birth defects. Although both maternal and inherited genotypes are thought to play a role in the etiology of CTDs, few specific genetic risk factors have been identified. To determine whether common variants acting through the genotype of the mother (e.g. via an in utero effect) or the case are associated with CTDs, we conducted a genome-wide association study of 750 CTD case-parent triads, with follow-up analyses in 358 independent triads. Log-linear analyses were used to assess the association of CTDs with the genotypes of both the mother and case. No association achieved genomewide significance in either the discovery or combined (discovery+follow-up) samples. However, three loci with p-values suggestive of association (p<10^−5^) in the discovery sample had p-values <0.05 in the follow-up sample and p-values in the combined data that were lower than in the discovery sample. These included suggestive association with an inherited intergenic variant at 20p12.3 (rs6140038, combined p = 1.0×10^−5^) and an inherited intronic variant in *KCNJ4* at 22q13.1 (rs2267386, combined p = 9.8×10^−6^), as well as with a maternal variant in *SLC22A24* at 11q12.3 (rs11231379, combined p = 4.2×10^−6^). These observations suggest novel candidate loci for CTDs, including loci that appear to be associated with the risk of CTDs via the maternal genotype, but further studies are needed to confirm these associations.

## Introduction

In the United States, birth defects are the leading cause of infant mortality [1], [2]. The most common birth defects are congenital heart defects, which occur in approximately 1% of live births and account for 40% of birth defect related deaths [3], [4]. Because heart defects include a wide range of conditions that may be etiologically heterogeneous, epidemiological studies generally focus on subgroups of these conditions for which there is evidence of a shared etiology [5]. Conotruncal and related malformations (CTDs) form one of the most common subgroups, accounting for approximately one-third of all congenital heart defects [6], [7].

Several lines of evidence suggest that the various CTD phenotypes (e.g. tetralogy of Fallot, conoventricular septal defects, d-transposition of the great arteries, double outlet right ventricle) share common genetic underpinnings [8], [9], [10], [11], [12]. For example, several different CTD phenotypes are observed among individuals with specific genetic syndromes (e.g., 22q11 deletion syndrome) [13], [14], [15]. In addition, family studies indicate that CTDs are highly heritable [7], [16], and that affected relatives of individuals with a CTD are more likely to have a CTD than other types of heart defects [17], [18], [19]. However, the genetic contribution to CTD risk is believed to be complex, perhaps involving both the maternal and inherited (i.e. case) genotypes [11], [12], [20], [21], [22], [23], and few specific genetic risk factors have been identified.

To identify genes that influence susceptibility to CTDs through the maternal and inherited (i.e. case) genotype, we conducted a family-based genome-wide association study (GWAS) and analyzed suggestive associations in an independent, family-based follow-up sample.

## Materials and Methods

### Ethics Statement

Study subjects provided consent under a protocol approved by the Children's Hospital of Philadelphia (CHOP) Institutional Review Board for the Protection of Human Subjects. Specifically, adult subjects provided written consent and parents or guardians provided written consent for minors.

### Study Subjects and Analysis

Case-parent triads were collected for a discovery sample between 1992–2010 at the Cardiac Center at the CHOP. Eligible diagnoses included: tetralogy of Fallot, D-transposition of the great arteries, ventricular septal defects (conoventricular, posterior malalignment and conoseptal hypoplasia), double outlet right ventricle, aortic arch anomalies, truncus arteriosus, and interrupted aortic arch. Diagnostic criteria have been previously described [24]. In particular, a conoventricular septal defect was defined as a defect in the interventricular septum that was located between a normally situated (i.e., not-malaligned) conal/infundibular septum and the muscular/trabecular septum, typically beneath part of the septal leaflet of the tricuspid valve. The diagnosis of a CTD in the case was confirmed by review of medical records. We performed fluorescence in situ hybridization (FISH) and/or multiplex ligation-dependent probe amplification using standard techniques to screen for 22q11 deletion syndrome when clinically suspected. Triads in which the case had a known chromosomal, genetic, or teratogenic syndrome, or in which the mother had type 1 or 2 diabetes, used insulin, or used an anticonvulsant during pregnancy were excluded since these conditions/exposures are known CTD risk factors.

Blood or saliva samples were collected from all CTD cases and their parents, and DNA extraction was performed using standard techniques (Puregene DNA isolation kit by Gentra Systems, Inc., Minneapolis, MN for blood samples, and Oragene DNA isolation kit by DNA Genotek Inc., Ontario, Canada for saliva samples). Genome-wide genotyping was performed at two time points using the Illumina HumanHap550 (v1, v3) and 610 BeadChip platforms, respectively, due to updates in the laboratory. Single nucleotide polymorphisms (SNPs) that were not represented on all BeadChips were excluded. Data for SNPs that met any of the following criteria were also excluded: (1) non-autosomal, (2) minor allele frequency <1%, (3) genotype distribution in parents deviated from Hardy-Weinberg equilibrium (p<1×10^−5^), (4) Mendelian error rate >1%, (5) call rate <95%. Data were also excluded for triads with a Mendelian error rate >1%, and for individuals with a genotype call rate <95%. Quality control analyses and exclusions were performed using PLINK v1.06 [25].

In the subset of triads in which both parents were non-Hispanic white by self-report, additional autosomal SNPs were imputed using MACH [26] version 1.0.16 and the phased HapMap II (release 22) CEU reference haplotypes (N = 60 founders). Imputed SNPs with imputation r^2^ (i.e., estimated squared correlation between the imputed and actual genotypes) <0.3 were excluded, as were all imputed SNPs with a MAF <1% or a Mendelian error rate >1%. To assess the accuracy of self-reported white race, we determined race using ancestry informative markers as described by Shaikh et al [27].

The associations between the maternal and inherited genotype for each variant and risk for CTDs were assessed using log-linear analyses [28], [29], [30], as implemented under the MI-GWAS platform [31]. Briefly, log-linear analysis has been widely used in genetic association studies of birth defects (e.g. [32], [33], [34], [35], [36]), and involves comparing the observed distribution of genotypes in the triads to the expected genotypes under the assumptions of both Mendelian inheritance and symmetry of maternal and parental genotypes [28], [29], [30]. Log-linear analysis has the advantage over the transmission disequilibrium test (TDT) of allowing for the evaluation of maternal as well as inherited genetic effects [28], [29], [30].

For each SNP, statistical significance was evaluated using a one-degree of freedom likelihood ratio test to compare a full model (including terms for both maternal and inherited genotypes) to a reduced model (excluding the parameter being tested). Using the default MI-GWAS parameters [31], an additive model was used for the genotype being tested (e.g., maternal genotype) and an unrestricted model was used for the other genotype (e.g., inherited genotype). We analyzed genotyped SNPs in the full GWAS dataset and both genotyped and imputed SNPs in the subset of non-Hispanic whites. Manhattan plots and q-q plots were constructed and lambda values were calculated using R version 2.15 (http://www.r-project.org/) for the full discovery cohort as well as the subset of non-Hispanic whites.

SNPs with p<10^−5^ were considered to have suggestive evidence of association with CTDs [37]. However, due to limitations on the number of variants that could be genotyped in the follow-up sample, we imposed additional criteria to select a subset of these SNPs for inclusion in the follow-up analysis. Specifically, each SNP for which the maternal or inherited genotype was associated with CTDs at p<10^−6^ was included in the follow-up study. In addition, select SNPs (described below) with association p-values 10^−6^<p<10^−5^ in either the full analytic group or the subgroup of non-Hispanic white triads were also included. The selected SNPs included those: with p<10^−5^ in both analytic groups; in regions with multiple associations at p<10^−5^; and in biologically plausible candidate genes (e.g, involved in pathways potentially related to heart development).

Additional, independent, predominantly white case-parent triad samples were collected for the follow-up sample, using the same criteria and methods as in the discovery sample. Genotyping of the follow-up sample was performed using a custom Illumina GoldenGate panel. A subset of samples from the discovery cohort was also genotyped using this platform, for comparisons with genotypes that were imputed in the discovery sample.

Quality control filters for SNPs genotyped in the follow-up sample were identical to those used for genotyped SNPs in the discovery sample. Data from the follow-up sample were analyzed using log linear analyses as described for the discovery sample. For SNPs with p<0.50 in the follow-up sample (and consistent directions of magnitudes of association between the discovery and follow-up samples), analyses were repeated in the combined (discovery + follow-up) sample.

For SNPs with combined p<10^−5^, we analyzed the predicted functional impact. We used Golden Helix SNP & Variation Suite v7.6 (Golden Helix, Inc., Bozeman, MT, www.goldenhelix.com) to annotate protein function scores (e.g., PolyPhen2) and the UCSC genome browser (hg19: genome.ucsc.edu) [38] to identify genes, transcription factor binding sites, and regions of open chromatin.

## Results

We recruited 852 case-parent triads for the discovery sample. After making exclusions based on the quality control criteria, there were 750 case-parent triads (1,868 individuals) in the discovery sample. The majority of the triads were Non-Hispanic white (n = 537 triads, 72%) (Table 1) and there was 99% concordance between self-reported white race and white classification by ancestry informative markers. The most frequent diagnoses among the cases were tetralogy of Fallot (39.2%), D-transposition of the great arteries (20.7%), and conoventricular septal defects (20.3%) (Table 1).

**Table 1:** Characteristics of cases with conotruncal and related heart defects.

	Discovery Sample		Follow-up Sample	
	N	(%)	N	(%)
Race/ethnicity				
Non-Hispanic white	537	71.6	348	97.2
Other	213	28.4	10	2.8
Sex				
Male	458	61.1	214	60.1
Female	292	38.9	142	39.9
Lesion				
Tetralogy of Fallot	294	39.2	119	33.4
D-transposition of the great arteries	155	20.7	76	21.4
Ventricular septal defects^A^	152	20.3	93	26.1
Double outlet right ventricle	80	10.7	22	6.2
Isolated aortic arch anomalies	34	4.5	21	5.9
Truncus arteriosus	20	2.7	15	4.2
Interrupted aortic arch	15	2.0	10	2.8
Total	750	(100.0)	358	(100.0)

A Includes conoventricular, posterior malalignment and conoseptal hypoplasia.

Log-linear analyses of the 530,347 genotyped SNPs that passed quality control criteria, in the full discovery cohort, identified nine maternal and eight inherited SNPs with suggestive (i.e., p<10^−5^) evidence of association [37] with CTDs, but none reached genome-wide significance (p<5×10^−8^) (Table S1). Analyses of the 530,347 genotyped and 1,890,943 imputed SNPs that passed quality control criteria (i.e., 2,421,290 total SNPs analyzed), in the non-Hispanic white triads, identified an additional 23 maternal and 80 inherited SNPs with suggestive evidence of association, but none reached genome-wide significance (Table S1, Figure S1). The q-q plots (Figure S2) suggested little deviation from expectation for maternal SNPs (lambda = 1.02 in the full analytic group and 1.00 in the non-Hispanic white subgroup) and minimal deviation from expectation for inherited SNPs (lambda = 1.08 for the full analytic group and 1.06 for the non-Hispanic white subgroup). Because tests involving the inherited genotype are not subject to bias due to population stratification in analyses of triad data [29], we did not attempt to reduce the genomic inflation factor.

Of the 32 maternal and 88 inherited genotypes with suggestive evidence of association with CTDs, 61 (see Materials and Methods for details of SNP selection) were assessed in the follow-up sample. Six of these 61 SNPs did not pass the genotyping quality control filters in the follow-up sample. Genotype data for the remaining 55 SNPs were available for 358, predominantly non-Hispanic white (97.2%, Table 1) triads in the follow-up sample. Log linear analyses of these data identified one maternal (rs11231379) and two inherited SNPs (rs6140038 and rs2267386) with p<0.05 in the follow-up sample (and consistent directions of magnitudes of association between the discovery and follow-up samples). In the combined analyses (discovery + follow-up samples), there was suggestive evidence of association (p<10^−5^) with each of these three variants and the combined p-values were less than the corresponding discovery p-values (Table 2). Several other maternal SNPs in the same region as rs11231379 were also nominally associated with CTDs in the discovery and follow-up samples (Table 2).

**Table 2:** Summary data for top variants with suggestive inherited or maternal association with conotruncal heart defects.

SNP	Chr^A^	Position^B^ (bp)	MAF^C^	Gene^D^	Function	Discover *P*-value	Follow-up *P*-value	Combine *P*-value	Discovery effect (95% CI)^E^	Follow-up effect (95% CI)^E^	Combined effect (95% CI)^E^
**Subgroup of non-Hispanic whites**											
**Maternal effects**											
rs11231379^F^	11	62661511	0.134	*SLC22A24*	Intron	4.70E-06	0.03	4.23E-06	1.88 (1.42–2.50)	1.73 (1.03–2.90)	1.85 (1.45–2.37)
**Inherited effects**											
rs6140038^F^	20	6530162	0.011	(AL121911.1)	Intergenic	3.69E-07	0.01	1.04E-06	24.79 (3.33–184.26)	3.33 (1.32–8.39)	5.24 (2.50–10.99)
rs2267386^F^	22	37162058	0.024	*KCNJ4*	Intron	2.23E-06	0.02	1.41E-06	4.79 (2.30–9.96)	2.43 (1.18–5.00)	3.12 (1.93–5.05)

A Chromosome.

B Hg18/NCBI build 36.

C Minor allele frequency among non-Hispanic white study participant founders (i.e. mother and father).

D For SNPs mapping within genes, gene names are listed, and for intergenic SNPs, the nearest gene is listed in parentheses.

E Relative risk estimate for carrying one copy of the high-risk allele compared to no copies, and corresponding 95% confidence interval.

F Imputed SNP; concordance between the imputed and assay-based genotypes in 21 samples from the discovery sample that were genotyped with the follow-up sample was 98.5%.

## Discussion

In the first reported GWAS of CTDs that included the evaluation of both inherited and maternal genetic effects, we identified several potentially interesting candidate regions for CTDs. Although no association achieved genome-wide significance (p<10^−8^), we report on several promising candidate regions, including loci associated with CTDs via the maternal genotype, that warrant further investigation.

**Figure 1:**
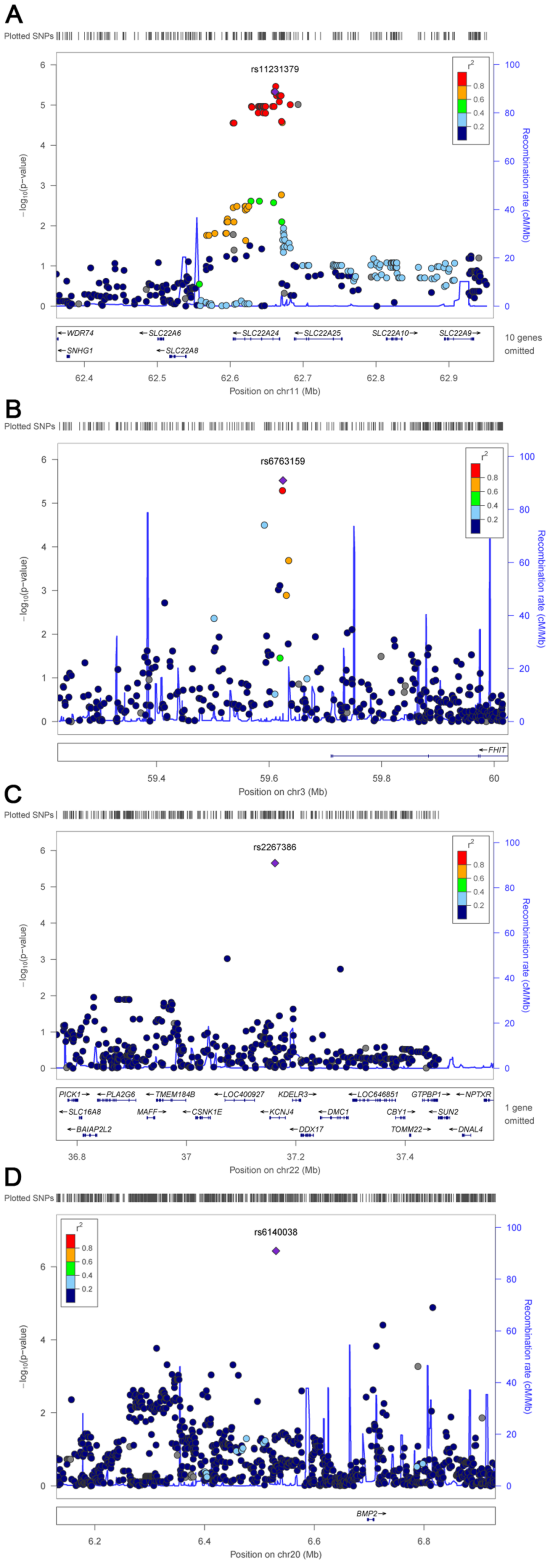
Loci showing suggestive associations with conotruncal malformations in the discovery sample. A) SNPs in *SLC22A24* B) SNPs near *FHIT* C) rs2267386 D) rs6140038. Each pane shows the log-linear model association statistic (−log_10_ p) on the left y axis for the discovery sample variant with the highest regional value that was confirmed in our follow-up sample (purple diamond) and nearby markers (circles). Linkage disequilibrium (r^2^) between this variant and nearby markers is indicated by red shading and recombination rates across each region in 1000 Genomes CEU data are indicated by blue lines on the right y axis. The position on the chromosome (hg18) and the position of nearby genes is shown on the x-axis.

There were seven maternal variants located in *SLC22A24* at 11q12.3 with suggestive evidence for association with CTDs (i.e. p<10^−5^) in the combined data (rs11231379, rs11231379, rs7948969, rs1939748, rs1939747, rs4393318, and rs4366490) (Table 2, Table S1). This gene encodes a transmembrane protein involved in organic ion transport across cellular membranes [39]. These SNPs are in strong linkage disequilibrium (r^2^>0.8), and include a missense mutation (rs1939748, Thr->Ser) that is fairly well-conserved [GERP++ [40] score: 2.3 and PhyloP [41] score: 1.3] and predicted to be “probably damaging” by PolyPhen2 [42]. An additional 30 maternal SNPs in this region, most of which are in tight linkage disequilibrium with these seven *SLC22A24* variants (r^2^>0.8), were also nominally associated with CTDs in the discovery sample (Figure 1a).

We also identified two relatively rare (MAF<5%) SNPs with suggestive evidence of association with CTDs via the inherited genotype. One of these SNPs (rs2267386) at 22q13.1 falls within an intron in *KCNJ4*, which encodes the inward rectifier potassium channel 4 protein (IRK4), a protein that is expressed in the fetal human heart and plays an important role in cardiac repolarization [43], [44], [45].

The other SNP with a suggestive inherited genetic effect, rs6140038, is intergenic and is located between *BMP2* (166 kb downstream) and *FERMT1* (477 kb upstream) at 20p12.3. *BMP2* is involved in differentiation of the secondary heart field progenitors into myocardium [46]. In animal models, *BMP2* is expressed by the primary outflow myocardium during the stages that the secondary myocardium is incorporated and induces expression of the contractile proteins in cells being incorporated into the outflow myocardium [47], [48]. The variant rs6140038 is flanked by two regions of open chromatin with corresponding CTCF sites (at 18 kb upstream, validated in GM12878 cells and K562 cells, and 164 kb downstream, validated in GM12878 cells), suggesting that it falls within a region of regulatory activity. *FERMT1* is involved in integrin signaling [49].

In the follow-up sample, there were 13 additional SNPs with p-values <0.50 for either the maternal or inherited genotype, consistent directions of association between the discovery and follow-up samples, and combined p-values that were suggestive of association (Table S1). These included maternal genotypes for two intergenic SNPs at 3p14.2 (rs6763159, rs1447807, Figure 1b, Table S1) that are in strong linkage disequilibrium (r^2^ = 1.0) and located approximately 86 kb downstream from *FHIT*, which encodes a tumor suppressor protein involved in cell cycle regulation and is expressed in fetal human cardiac tissue [45], [50], [51]. There are several validated regions of open chromatin upstream of these SNPs (approximately 613 kb, 451 kb, 350 kb, and 88 kb upstream of rs6763159), many of which coincide with validated transcription factor binding sites (e.g., PolII site at 613 kb; CTCF sites at 610 kb, 451 kb, 350 kb, and 88 kb; an NFKB site at 352 kb; and FOXA1, FOXA2, GATA3, and CEBPB sites at 9 kb). These findings suggest that the upstream regions of open chromatin may have regulatory activity.

Cordell et al. recently published a case-control GWAS of tetralogy of Fallot, the most common CTD among our cases [52]. Associations between inherited genotypes and tetralogy of Fallot were reported for a region on chromosome 12q24 (six SNPs) and 13q32 (two SNPs) [52]; however, the inherited genotypes for these eight SNPs were not associated with CTDs in our data (range of p-values for these eight SNPs among our non-Hispanic white triads analyses: 0.54–0.94). Cordell et al. did not evaluate any of the SNPs that were associated with CTDs via the inherited genotype in our study (i.e., those listed in Table 2) or SNPs in tight linkage disequilibrium with these SNPs. Further, they did not evaluate association with the maternal genotype. However, they did evaluate the inherited genotype for the two SNPs near *FHIT* for which we found suggestive evidence of an association via the maternal genotype; they reported p-values for these SNPs that were even lower than those in our follow-up sample (rs6763159 p = 0.0006, odds ratio  = 0.83; rs1447807 p = 0.0008, odds ratio  = 0.83). Since the inherited genotype is confounded with the maternal genotype in case-control studies [29], this provides some limited additional support for an association, via the maternal genotype, between this region and the risk of CTDs. There was no overlap between the regions with suggestive evidence of association with CTDs in our study and top loci from other recent GWAS or genome-wide linkage analyses of heart defects [53], [54], [55].

Although our analyses were limited by a relatively small sample, given the rarity of CTDs, our sample represents one of the largest. However, it was not possible to analyze specific types of defects, and we cannot rule out the possibility that our analyses of all CTDs could have missed loci associated with specific defects. This study is one of the first GWAS of any disease to identify suggestive associations with maternal genetic regions, and our results emphasize that accounting for maternal genetic effects in GWAS may broaden our understanding of the genetics of complex traits, particularly traits with a young age of onset.

## Supporting Information

Figure S1 Manhattan plot of log-linear model likelihood ratio test p-values (on logarithmic scale) in the discovery cohort for A) inherited genotyped SNPs in the full analytic group B) maternal genotyped SNPs in the full analytic group C) inherited genotyped and imputed SNPs in the non-Hispanic white subgroup D) maternal genotyped and imputed SNPs in the Non-Hispanic white subgroup.
(TIF)

Figure S2 Quantile-quantile plot showing observed (black dots) and expected p-values (orange line) for the log-linear model likelihood ratio tests in the discovery cohort for A) inherited genotyped SNPs in the full analytic group (lambda = 1.08) B) maternal genotyped SNPs in the full analytic group (lambda = 1.02) C) inherited genotyped and imputed SNPs in the non-Hispanic white subgroup (lambda = 1.06) D) maternal genotyped and imputed SNPs in the non-Hispanic white subgroup (lambda = 1.00).
(TIF)

Table S1 Summary data for all variants with suggestive inherited or maternal association with conotruncal heart defects in the discovery sample.
(DOC)
