# Supplementary figures and images for: Genome-Wide Association Study of Maternal and Inherited Loci for Conotruncal Heart Defects
Source: PLoS One. 2014 May 6;9(5):e96057. doi: 10.1371/journal.pone.0096057 (PMC4011736; doi:10.1371/journal.pone.0096057)

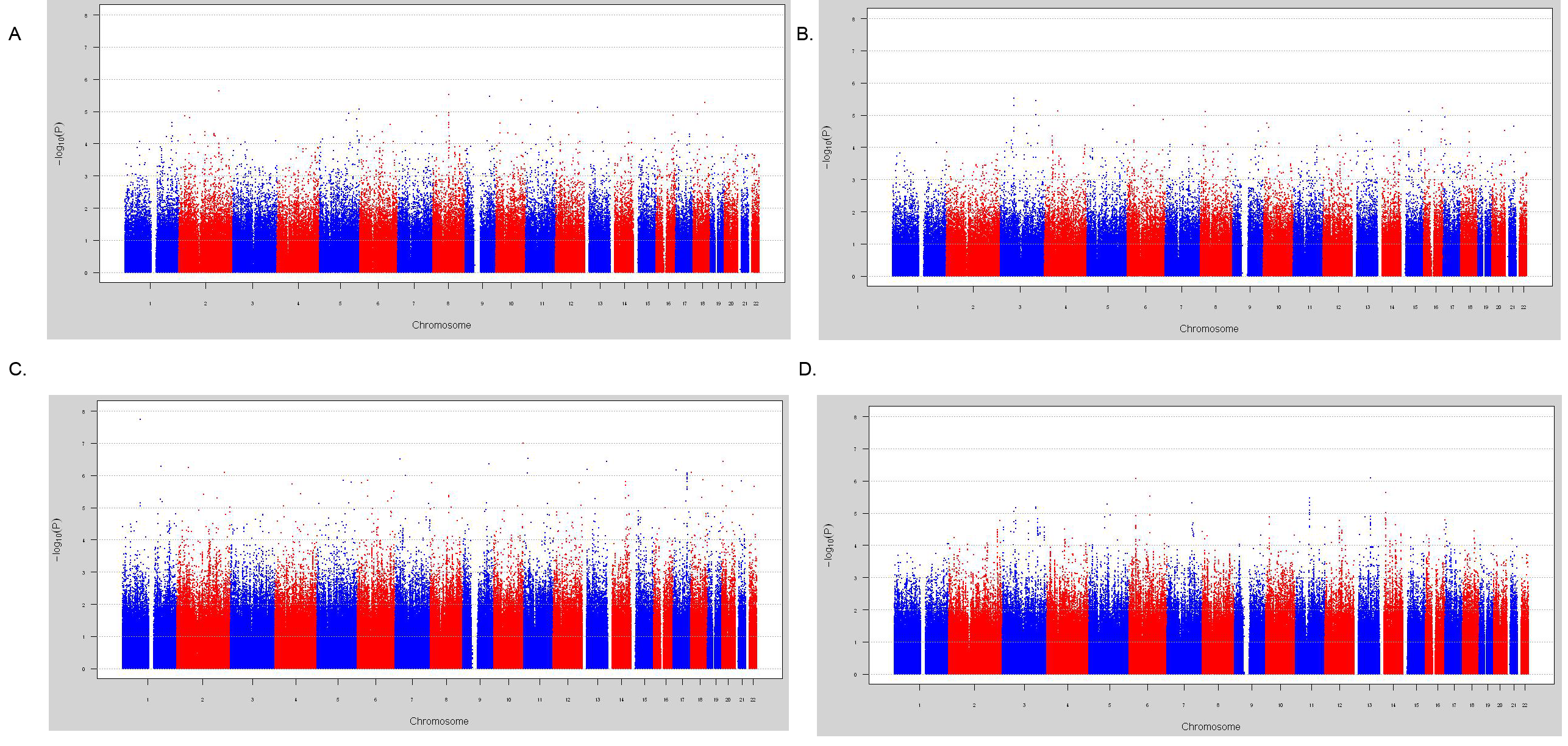

Supplement: Figure S1 — Manhattan plot of log-linear model likelihood ratio test p-values (on logarithmic scale) in the discovery cohort for A) inherited genotyped SNPs in the full analytic group B) maternal genotyped SNPs in the full analytic group C) inherited genotyped and imputed SNPs in the non-Hispanic white subgroup D) maternal genotyped and imputed SNPs in the Non-Hispanic white subgroup. (TIF) [file pone.0096057.s001.tif]

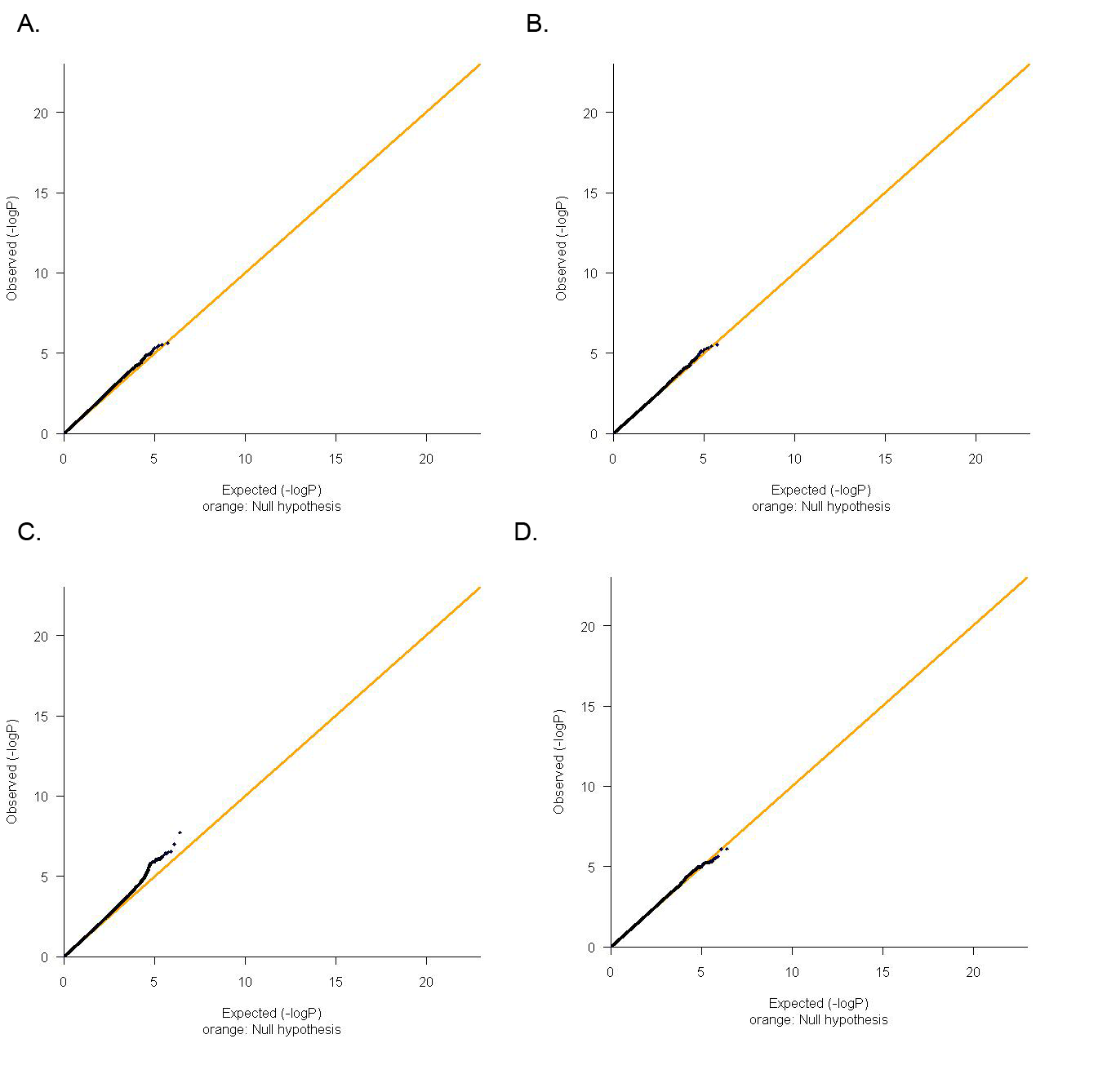

Supplement: Figure S2 — Quantile-quantile plot showing observed (black dots) and expected p-values (orange line) for the log-linear model likelihood ratio tests in the discovery cohort for A) inherited genotyped SNPs in the full analytic group (lambda = 1.08) B) maternal genotyped SNPs in the full analytic group (lambda = 1.02) C) inherited genotyped and imputed SNPs in the non-Hispanic white subgroup (lambda = 1.06) D) maternal genotyped and imputed SNPs in the non-Hispanic white subgroup (lambda = 1.00). (TIF) [file pone.0096057.s002.tif]
