# Supplementary material for: Genome-Wide Association Study of Maternal and Inherited Loci for Conotruncal Heart Defects
Source: PLoS One. 2014 May 6;9(5):e96057. doi: 10.1371/journal.pone.0096057 (PMC4011736; doi:10.1371/journal.pone.0096057)
Supplement: Table S1 — Summary data for all variants with suggestive inherited or maternal association with conotruncal heart defects in the discovery sample. (DOC) [file pone.0096057.s003.doc]

Table S1. Summary data for all variants with suggestive inherited or maternal association with conotruncal heart defects in the discovery sample.

| SNP | Chr^A^ | Position^B^ (bp) | MAF^C^ | Gene^D^ | Function | Discovery  *P*-value^E^ | Follow-up  *P*-value^F^ | Combined  *P*-value | Discovery effect  (95% CI)^G^ | Follow-up effect  (95% CI)^G^ | Combined effect  (95% CI)^G^ |
| --- | --- | --- | --- | --- | --- | --- | --- | --- | --- | --- | --- |
| **Full analytic group** |  |  |  |  |  |  |  |  |  |  |  |
| **Inherited** |  |  |  |  |  |  |  |  |  |  |  |
| rs1546813 | 2 | 178582028 | 0.272 | *PDE11A* | intron | 2.41E-06 | - |  | 1.56 (1.30-1.89) |  |  |
| rs6897795 | 5 | 177539025 | 0.141 | *(HNRNPAB)* | intergenic | 8.55E-06 | - |  | 1.75 (1.35-2.22) |  |  |
| rs7010162 | 8 | 71139059 | 0.439 | *PRDM14* | intron | 3.04E-06 | - |  | 1.49 (1.27-1.75) |  |  |
| rs6477693 | 9 | 110958186 | 0.295 | *C9orf4* | intron | 3.53E-06 | 0.85 |  | 1.56 (1.28-1.89) |  |  |
| rs2804591 | 10 | 113777559 | 0.182 | *(GPAM)* | intergenic | 2.99E-06 | - |  | 1.74 (1.37-2.20) |  |  |
| rs12275681 | 11 | 119679055 | 0.094 | *POU2F3* | intron | 4.76E-06 | - |  | 2.00 (1.47-2.70) |  |  |
| rs9538184 | 13 | 58221736 | 0.207 | *(DIAPH3)* | intergenic | 8.61E-06 | - |  | 1.56 (1.28-1.92) |  |  |
| rs784240 | 18 | 51585120 | 0.062 | *(TCF4)* | intergenic | 5.65E-06 | - |  | 2.36 (1.59-3.50) |  |  |
| **Maternal** |  |  |  |  |  |  |  |  |  |  |  |
| rs1447807 | 3 | 59623721 | 0.458 | *(FHIT)* | Intergenic | 5.15E-06 | 0.17 | 2.56E-06 | 0.67 (0.56-0.80) | 0.78 (0.54-1.12) | 0.69 (0.59-0.81) |
| rs6763159 | 3 | 59624561 | 0.469 | *(FHIT)* | intergenic | 3.03E-06 | 0.16 | 1.41E-06 | 0.66 (0.56-0.79) | 0.77 (0.54-1.11) | 0.68 (0.58-0.80) |
| rs9827048 | 3 | 160467767 | 0.175 | *(IQCJ)* | intergenic | 3.44E-06 | - |  | 1.67 (1.33-2.08) |  |  |
| rs1448996 | 3 | 160475933 | 0.143 | *SCHIP1* | intron | 9.92E-06 | - |  | 1.67 (1.32-2.12) |  |  |
| rs1480303 | 4 | 58487650 | 0.216 | *(IGFBP7)* | Intergenic | 7.57E-06 | - |  | 1.60 (1.29-1.96) |  |  |
| rs2844660 | 6 | 30931739 | 0.094 | *(DDR1)* | Intergenic | 5.18E-06 | 1.00 |  | 1.92 (1.43-2.56) |  |  |
| rs12682168 | 8 | 22723667 | 0.204 | *PEBP4* | intron | 7.77E-06 | - |  | 1.56 (1.28-1.91) |  |  |
| rs11638711 | 15 | 34359440 | 0.350 | *(C15orf41)* | Intergenic | 8.36E-06 | - |  | 1.49 (1.25-1.79) |  |  |
| rs11117451 | 16 | 84720722 | 0.348 | *(IRF8)* | Intergenic | 5.53E-06 | - |  | 1.49 (1.25-1.75) |  |  |
| **Subgroup^H^** |  |  |  |  |  |  |  |  |  |  |  |
| **Inherited** |  |  |  |  |  |  |  |  |  |  |  |
| rs6686802^I^ | 1 | 81144477 | 0.033 | (AC091613.1) | Intergenic | 1.86E-08 | 0.40 | 0.002 | 5.18 (2.69-9.98) | 1.29 (0.71-2.37) | 1.80 (1.23-2.64) |
| rs12136445^I^ | 1 | 82816827 | 0.046 | (AC093580.1) | Intergenic | 9.04E-06 | 0.49 |  | 2.82 (1.74-4.57) | 0.79 (0.41-1.54) |  |
| rs17101444^I^ | 1 | 83427965 | 0.061 | LOC100288807 | Intron | 7.04E-06 | - |  | 2.44 (1.63-3.65) |  |  |
| rs16847585^I^ | 1 | 173113605 | 0.017 | *RABGAP1L* | Intron | 5.45E-06 | - |  | 7.48 (2.60-21.55) |  |  |
| rs2130576^I^ | 1 | 177447725 | 0.044 | *ABL2* | Intron | 5.32E-07 | Failed |  | 3.45 (2.04-5.88) |  |  |
| rs17483344^I^ | 1 | 181539971 | 0.027 | *NMNAT2* | Intron | 6.50E-06 | - |  | 3.85 (2.04-7.69) |  |  |
| rs6545278^I^ | 2 | 52559556 | 0.048 | (AC139712.2) | Intergenic | 5.84E-07 | 0.11 | 7.86E-07 | 3.28 (1.98-5.43) | 1.82 (0.87-3.82) | 2.62 (1.76-3.90) |
| rs13026971^I^ | 2 | 119923217 | 0.045 | *SCTR* | Intron | 3.92E-06 | - |  | 2.86 (1.79-4.55) |  |  |
| rs12151759^I^ | 2 | 181351449 | 0.081 | (AC074098.1) | Intergenic | 5.05E-06 | - |  | 2.13 (1.54-3.03) |  |  |
| rs765161^I^ | 2 | 215483281 | 0.052 | *(ABCA12)* | Intergenic | 9.79E-07 | 0.99 |  | 2.90 (1.84-4.57) |  |  |
| rs12612849^I^ | 2 | 240107639 | 0.079 | (AC079612.1) | Intergenic | 9.83E-06 | 0.24 | 1.11E-05 | 2.16 (1.53-3.10) | 1.39 (0.81-2.40) | 1.87 (1.41-2.48) |
| rs6706785^I^ | 2 | 240108990 | 0.079 | (AC079612.1) | Intergenic | 9.84E-06 | 0.18 | 6.09E-06 | 2.16 (1.52-3.07) | 1.47 (0.84-2.56) | 1.91 (1.44-2.54) |
| rs17512490^I^ | 4 | 40086573 | 0.215 | (AC098869.2) | Intergenic | 8.97E-06 | - |  | 1.69 (1.33-2.13) |  |  |
| rs13116700^I^ | 4 | 79362651 | 0.016 | *FRAS1* | Intron | 2.27E-06 | 0.53 |  | 10.00 (2.78-33.33) |  |  |
| rs11734905^I^ | 4 | 115732689 | 0.017 | *(UGT8)* | Intergenic | 3.69E-06 | - |  | 6.67 (2.56-16.67) |  |  |
| rs17797426^I^ | 5 | 6919701 | 0.037 | (AC122710.1) | Intergenic | 7.43E-06 | - |  | 3.23 (1.85-5.56) |  |  |
| rs10069047^I^ | 5 | 117547841 | 0.049 | (AC122716.1) | Intergenic | 1.41E-06 | - |  | 2.92 (1.84-4.62) |  |  |
| rs17612186^I^ | 5 | 141869254 | 0.210 | (AC005592.1) | Intergenic | 7.31E-06 | - |  | 1.68 (1.34-2.12) |  |  |
| rs961547^I^ | 5 | 154590136 | 0.023 | (AC010591.1) | Intergenic | 1.63E-06 | 0.63 |  | 4.85 (2.34-10.05) |  |  |
| rs12174402^I^ | 6 | 20522984 | 0.093 | *E2F3* | Intron | 1.79E-06 | 0.92 |  | 2.18 (1.57-3.02) |  |  |
| rs9381631 | 6 | 47996659 | 0.224 | *C6orf138* | Intron | 1.48E-06 | - |  | 1.74 (1.38-2.18) |  |  |
| rs9349436^I^ | 6 | 47997303 | 0.216 | *C6orf138* | Intron | 4.60E-06 | - |  | 1.69 (1.38-2.13) |  |  |
| rs9495200^I^ | 6 | 139037155 | 0.025 | *(NHSL1)* | Intergenic | 6.36E-06 | - |  | 4.55 (2.17-9.09) |  |  |
| rs610231^I^ | 6 | 154420170 | 0.117 | *OPRM1* | Intron | 5.05E-06 | - |  | 1.96 (1.47-2.63) |  |  |
| rs794104^I^ | 6 | 164605325 | 0.036 | (AL358972.1) | Intergenic | 3.08E-06 | - |  | 3.40 (1.95-5.90) |  |  |
| rs940433^I^ | 7 | 21682393 | 0.039 | *DNAH11* | Intron | 3.12E-07 | 0.04 | 2.97E-05 | 3.61 (2.12-6.14) | 1.65 (1.04-2.64) | 2.02 (1.45-2.81) |
| rs17172851^I^ | 7 | 47323622 | 0.014 | *TNS3* | Intron | 1.01E-06 | - |  | 14.29 (3.23-50.00) |  |  |
| rs4071539^I^ | 7 | 152151775 | 0.052 | *ACTR3B* | Intron | 6.40E-06 | - |  | 2.66 (1.70-4.15) |  |  |
| rs11778473^I^ | 8 | 5359757 | 0.027 | (AC091193.1) | Intergenic | 1.67E-06 | - |  | 4.35 (2.22-8.33) |  |  |
| rs4739605^I^ | 8 | 81734103 | 0.019 | *ZNF704* | Intron | 4.68E-06 | - |  | 5.88 (2.38-14.29) |  |  |
| rs1153029^I^ | 8 | 81941055 | 0.019 | *ZNF704* | Intron | 4.33E-06 | - |  | 5.88 (2.44-14.29) |  |  |
| rs2069135^I^ | 8 | 93626943 | 0.028 | (AC091096.1) | Intergenic | 9.70E-06 | - |  | 3.66 (1.96-6.84) |  |  |
| rs6477693 | 9 | 110958186 | 0.286 | *C9orf4* | Intron | 7.36E-06 | 0.97 |  | 1.64 (1.32-2.04) |  |  |
| rs436582^I^ | 9 | 117996489 | 0.032 | *PAPPA* | Intron | 4.38E-07 | 0.10 | 3.16E-06 | 4.17 (2.27-7.69) | 1.79 (0.90-3.58) | 2.64 (1.73-4.03) |
| rs17232877^I^ | 10 | 107462933 | 0.040 | (RP11-45P22.1) | Intergenic | 8.80E-06 | - |  | 2.91 (1.77-4.77) |  |  |
| rs11017328^I^ | 10 | 132237090 | 0.042 | (AL355500.2) | Intergenic | 9.90E-08 | 0.31 | 2.85E-07 | 3.86 (2.23-6.68) | 1.52 (0.68-3.44) | 2.87 (1.88-4.40) |
| rs3959929^I^ | 11 | 15761710 | 0.061 | *(SOX6)* | Intergenic | 8.41E-07 | 0.67 |  | 2.72 (1.79-4.14) |  |  |
| rs17298726^I^ | 11 | 21048301 | 0.026 | *NELL1* | Intron | 2.96E-07 | 0.69 |  | 5.26 (2.50-11.11) |  |  |
| rs11213230^I^ | 11 | 109377633 | 0.033 | (RP11-361A21.1) | Intergenic | 7.36E-06 | - |  | 3.33 (1.89-5.88) |  |  |
| rs12581234^I^ | 12 | 115563054 | 0.114 | *(MAP1LC3B2)* | Intergenic | 1.67E-06 | - |  | 2.04 (1.52-2.78) |  |  |
| rs3851660^I^ | 12 | 126289885 | 0.025 | (AC079949.1) | Intergenic | 8.66E-06 | - |  | 4.21 (2.09-8.51) |  |  |
| rs9506979^I^ | 13 | 22434335 | 0.043 | (AL157931.2) | Intergenic | 5.52E-07 | Failed QC |  | 3.52 (2.06-6.02) |  |  |
| rs7986849^I^ | 13 | 58208059 | 0.235 | (AL354807.1) | Intergenic | 5.36E-06 | - |  | 1.67 (1.33-2.09) |  |  |
| rs9555453^I^ | 13 | 107746654 | 0.044 | *TNFSF13B* | Intron | 3.62E-07 | Failed |  | 3.48 (2.07-5.87) |  |  |
| rs8013100^I^ | 14 | 79969722 | 0.021 | *(C14orf145)* | Intergenic | 2.00E-06 | - |  | 5.56 (2.44-12.50) |  |  |
| rs1571381^I^ | 14 | 81278787 | 0.024 | (AL160192.2) | Intergenic | 5.12E-06 | 0.22 | 2.84E-06 | 4.66 (2.20-9.86) | 1.84 (0.70-4.80) | 3.53 (2.01-6.20) |
| rs1959122^I^ | 14 | 81407418 | 0.013 | (AL160192.2) | Intergenic | 2.37E-06 | 0.41 | 8.54E-07 | 9.45 (2.84-31.37) | 2.47 (0.33-18.60) | 7.63 (2.92-19.95) |
| rs12892504^I^ | 14 | 93555168 | 0.037 | *(OTUB2)* | Intergenic | 4.24E-06 | - |  | 3.23 (1.89-5.26) |  |  |
| rs12150130^I^ | 17 | 14058007 | 0.024 | (AC005224.1) | Intergenic | 6.73E-07 | 0.27 | 6.12E-06 | 5.38 (2.50-11.57) | 1.83 (0.25-13.39) | 3.48 (1.96-6.21) |
| rs11655826^I^ | 17 | 58195480 | 0.030 | *MARCH10* | Intron | 5.96E-06 | - |  | 4.13 (2.09-8.15) |  |  |
| rs7211558 | 17 | 61812941 | 0.155 | *PRKCA* | Intron | 2.30E-06 | 0.07 |  | 1.90 (1.44-2.51) | 0.73 (0.52-1.02) |  |
| rs4536508 | 17 | 61813793 | 0.157 | *PRKCA* | Intron | 1.34E-06 | 0.03 |  | 1.92 (1.46-2.53) | 0.69 (0.49-0.97) |  |
| rs4281768^I^ | 17 | 61814106 | 0.160 | *PRKCA* | Intron | 2.79E-06 | 0.12 |  | 1.90 (1.44-2.51) | 0.75 (0.53-1.07) |  |
| rs4569323^I^ | 17 | 61814190 | 0.163 | *PRKCA* | Intron | 1.24E-06 | 0.11 |  | 1.93 (1.47-2.54) | 0.75 (0.53-1.07) |  |
| rs7218479^I^ | 17 | 61814680 | 0.163 | *PRKCA* | Intron | 1.24E-06 | 0.02 |  | 1.93 (1.47-2.54) | 0.67 (0.48-0.94) |  |
| rs7208898^I^ | 17 | 61815948 | 0.163 | *PRKCA* | Intron | 1.24E-06 | 0.05 |  | 1.93 (1.47-2.54) | 0.71 (0.50-1.00) |  |
| rs4791063^I^ | 17 | 61816265 | 0.163 | *PRKCA* | Intron | 1.24E-06 | 0.10 |  | 1.93 (1.47-2.54) | 0.75 (0.54-1.06) |  |
| rs8071564^I^ | 17 | 61817089 | 0.160 | *PRKCA* | Intron | 2.79E-06 | 0.06 |  | 1.90 (1.44-2.51) | 0.72 (0.51-1.02) |  |
| rs6504419^I^ | 17 | 61817149 | 0.161 | *PRKCA* | Intron | 2.65E-06 | 0.09 |  | 1.90 (1.44-2.50) | 0.74 (0.52-1.05) |  |
| rs6504423^I^ | 17 | 61817531 | 0.126 | *PRKCA* | Intron | 1.24E-06 | 0.11 |  | 1.93 (1.47-2.54) | 0.75 (0.53-1.07) |  |
| rs9897870^I^ | 17 | 61820776 | 0.163 | *PRKCA* | Intron | 1.24E-06 | 0.14 |  | 1.93 (1.47-2.54) | 0.76 (0.54-1.09) |  |
| rs8073750^I^ | 17 | 61820950 | 0.163 | *PRKCA* | Intron | 2.79E-06 | 0.10 |  | 1.90 (1.44-2.51) | 0.73 (0.51-1.05) |  |
| rs8074703^I^ | 17 | 61821519 | 0.163 | *PRKCA* | Intron | 1.24E-06 | 0.15 |  | 1.93 (1.47-2.54) | 0.77 (0.54-1.09) |  |
| rs8075733^I^ | 17 | 61821640 | 0.163 | *PRKCA* | Intron | 1.24E-06 | 0.39 |  | 1.93 (1.47-2.54) | 0.86 (0.60-1.22) |  |
| rs4261587^I^ | 17 | 61824672 | 0.126 | *PRKCA* | Intron | 9.20E-07 | 0.23 |  | 2.13 (1.55-2.91) | 0.79 (0.55-1.15) |  |
| rs8082587^I^ | 17 | 61827936 | 0.126 | *PRKCA* | Intron | 9.20E-07 | 0.32 |  | 2.13 (1.55-2.91) | 0.82 (0.56-1.20) |  |
| rs6504425^I^ | 17 | 61828086 | 0.126 | *PRKCA* | Intron | 9.20E-07 | 0.27 |  | 2.13 (1.55-2.91) | 0.80 (0.55-1.17) |  |
| rs6504426^I^ | 17 | 61828161 | 0.126 | *PRKCA* | Intron | 9.20E-07 | 0.27 |  | 2.13 (1.55-2.91) | 0.81 (0.56-1.18) |  |
| rs4417582^I^ | 17 | 61831249 | 0.126 | *PRKCA* | Intron | 8.48E-07 | 0.22 |  | 2.13 (1.55-2.92) | 0.79 (0.54-1.14) |  |
| rs4417581 | 17 | 61831270 | 0.120 | *PRKCA* | Intron | 9.28E-07 | Failed |  | 2.13 (1.54-2.86) |  |  |
| rs7222507^I^ | 17 | 61836619 | 0.126 | *PRKCA* | Intron | 8.48E-07 | 0.38 |  | 2.13 (1.55-2.92) | 0.84 (0.57-1.23) |  |
| rs4630585^I^ | 17 | 61839105 | 0.125 | *PRKCA* | Intron | 1.14E-06 | 0.36 |  | 2.11 (1.54-2.90) | 1.20 (0.82-1.75) |  |
| rs4624218^I^ | 17 | 61839555 | 0.125 | *PRKCA* | Intron | 1.54E-06 | 0.28 |  | 2.10 (1.53-2.87) | 0.81 (0.56-1.18) |  |
| rs11872184^I^ | 18 | 2729733 | 0.018 | *SMCHD1* | Intron | 8.13E-07 | 0.25 | 4.32E-06 | 8.31 (2.91-23.75) | 1.84 (0.67-5.10) | 4.08 (2.15-7.74) |
| rs17558483^I^ | 18 | 50227128 | 0.023 | *(C18orf54)* | Intergenic | 4.59E-06 | - |  | 4.76 (2.22-10.00) |  |  |
| rs17694975^I^ | 18 | 54779664 | 0.035 | *ZNF532* | Intron | 1.46E-06 | - |  | 3.75 (2.08-6.74) |  |  |
| rs11696445^I^ | 20 | 2794252 | 0.022 | *PTPRA* | Intron | 2.15E-06 | - |  | 5.10 (2.36-11.01) |  |  |
| rs6140038^I^ | 20 | 6530162 | 0.011 | (AL121911.1) | Intergenic | 3.69E-07 | 0.01 | 1.04E-06 | 24.79 (3.33-184.26) | 3.33 (1.32-8.39) | 5.24 (2.50-10.99) |
| rs10446037^I^ | 20 | 19364489 | 0.024 | *SLC24A3* | Intron | 9.00E-06 | - |  | 4.17 (2.08-8.33) |  |  |
| rs230019^I^ | 20 | 49178602 | 0.074 | *(RPSAP1)* | Intergenic | 3.16E-06 | - |  | 2.33 (1.61-3.36) |  |  |
| rs10470148^I^ | 21 | 28387891 | 0.045 | (AL035610.1) | Intergenic | 1.26E-06 | - |  | 3.17 (1.92-5.23) |  |  |
| rs2267386^I^ | 22 | 37162058 | 0.024 | *KCNJ4* | Intron | 2.23E-06 | 0.02 | 1.41E-06 | 4.79 (2.30-9.96) | 2.43 (1.18-5.00) | 3.12 (1.93-5.05) |
| **Maternal** |  |  |  |  |  |  |  |  |  |  |  |
| rs4522758^I^ | 3 | 51891225 | 0.143 | *(IQCF5)* | Intergenic | 8.76E-06 | - |  | 1.85 (1.39-2.44) |  |  |
| rs6787031^I^ | 3 | 59622197 | 0.473 | *(FHIT)* | Intergenic | 7.14E-06 | Failed QC |  | 1.57 (1.28-1.92) |  |  |
| rs939249^I^ | 3 | 150331802 | 0.317 | *HPS3* | Intron | 6.51E-06 | 0.99 |  | 1.54 (1.27-1.89) |  |  |
| rs2331536^I^ | 3 | 150331953 | 0.317 | *HPS3* | Intron | 6.51E-06 | - |  | 1.54 (1.27-1.89) |  |  |
| rs4681169 | 3 | 150335145 | 0.326 | *HPS3* | Intron | 6.92E-06 | 0.89 |  | 1.54 (1.27-1.89) |  |  |
| rs10044415^I^ | 5 | 81987777 | 0.065 | (AC008885.1) | Intergenic | 5.24E-06 | - |  | 2.50 (1.64-3.85) |  |  |
| rs11749713 | 5 | 81994409 | 0.062 | (LOC92270) | Intergenic | 5.26E-06 | - |  | 2.50 (1.64-3.85) |  |  |
| rs2844660 | 6 | 30931739 | 0.110 | *(DDR1)* | Intergenic | 7.69E-07 | 0.90 |  | 2.17 (1.59-3.03) |  |  |
| rs11751768^I^ | 6 | 93753045 | 0.316 | (AL138731.1) | Intergenic | 2.95E-06 | - |  | 1.67 (1.33-2.08) |  |  |
| rs1450832 | 7 | 113650620 | 0.286 | *(FOXP2)* | Intergenic | 4.85E-06 | - |  | 1.64 (1.32-2.04) |  |  |
| rs1939757^I^ | 11 | 62660399 | 0.140 | *SLC22A24* | Intron | 4.70E-06 | Failed |  | 1.88 (1.42-2.50) |  |  |
| rs11231379^I^ | 11 | 62661511 | 0.134 | *SLC22A24* | Intron | 4.70E-06 | 0.03 | 4.23E-06 | 1.88 (1.42-2.50) | 1.73 (1.03-2.90) | 1.85 (1.45-2.37) |
| rs7106537^I^ | 11 | 62662417 | 0.140 | *SLC22A24* | Intron | 3.47E-06 | - |  | 1.89 (1.43-2.50) |  |  |
| rs7948969^I^ | 11 | 62663624 | 0.167 | *SLC22A24* | Intron | 5.87E-06 | 0.10 | 1.66E-06 | 1.75 (1.36-2.25) | 1.47 (0.93-2.32) | 1.69 (1.35-2.10) |
| rs1939749 | 11 | 62667425 | 0.166 | *SLC22A25* | Intron | 8.51E-06 | - |  | 1.73 (1.35-2.22) |  |  |
| rs1939748^I^ | 11 | 62667655 | 0.167 | *SLC22A24* | Missense | 5.87E-06 | 0.08 | 1.41E-06 | 1.75 (1.36-2.25) | 1.49 (0.94-2.35) | 1.69 (1.36-2.11) |
| rs1939747^I^ | 11 | 62668234 | 0.167 | *SLC22A24* | 5’ | 5.87E-06 | 0.10 | 1.82E-06 | 1.75 (1.36-2.25) | 1.44 (0.92-2.28) | 1.68 (1.35-2.09) |
| rs4393318^I^ | 11 | 62669361 | 0.167 | *SLC22A24* | 5’ | 5.87E-06 | 0.10 | 1.72E-06 | 1.75 (1.36-2.25) | 1.46 (0.93-2.29) | 1.68 (1.35-2.09) |
| rs4366490^I^ | 11 | 62669430 | 0.167 | *SLC22A24* | 5’ | 5.87E-06 | 0.08 | 1.30E-06 | 1.75 (1.36-2.25) | 1.51 (0.95-2.40) | 1.70 (1.36-2.12) |
| rs10736724^I^ | 11 | 62669834 | 0.167 | *SLC22A24* | 5’ | 6.03E-06 | - |  | 1.75 (1.37-2.27) |  |  |
| rs12364655^I^ | 11 | 62682384 | 0.127 | *(SLC22A25)* | Intergenic | 9.82E-06 | - |  | 1.92 (1.43-2.63) |  |  |
| rs11231390^I^ | 11 | 62693106 | 0.136 | *SLC22A25* | Intron | 9.74E-06 | - |  | 1.85 (1.39-2.44) |  |  |
| rs9593012^I^ | 13 | 74109874 | 0.122 | (AL157814.1) | Intergenic | 7.94E-07 | 0.53 |  | 2.16 (1.56-2.98) | 0.99 (0.64-1.53) |  |
| rs1245314^I^ | 14 | 26562480 | 0.369 | (AL110292.1) | Intergenic | 2.30E-06 | - |  | 1.59 (1.30-1.94) |  |  |

^A^ Chromosome

^B^ Hg18/NCBI build 36

^C^ Minor allele frequency among non-Hispanic Caucasian study participant founders (i.e. mother and father)

^D^ For SNPs mapping within genes, gene names are listed, and for intergenic SNPs, the nearest gene is listed in parentheses

^E^ *P*-values only shown for top inherited or maternal effects (*p*<1 x 10^-5^)

^F^ Replication criteria are provided in the Methods section of the manuscript

^G^ Relative risk estimate for carrying one copy of the high risk allele compared to no copies, and corresponding 95% confidence interval

^H^ Subgroup of non-Hispanic Caucasians

^I^ Imputed SNP
